# Drying Temperature Dictates Ileal Amino Acid Digestibility of Enzyme-Treated Soybean Meal in 25 kg Pigs

**DOI:** 10.3390/ani15223288

**Published:** 2025-11-13

**Authors:** Xianyi Tan, Chao Liu, Lixuan Lu, Yong Zhuo, Lin Li, Yunxiang Liang

**Affiliations:** 1 College of Life Science and Technology, Huazhong Agricultural University, Wuhan 430070, China; tanxianyi1981@163.com; 2 National Key Laboratory of Agricultural Microbiology, Huazhong Agricultural University, Wuhan 430070, China; 3 Bioengineering Institute, Sichuan Runge Biotechnology Co., Ltd., Deyang 618200, China; 4 Animal Nutrition Institute, Sichuan Agricultural University, Chengdu 611130, China; zhuoyong@sicau.edu.cn

**Keywords:** air-drying temperature, enzyme-treated soybean meal, ileal amino acid digestibility, fishmeal, pigs

## Abstract

Soybean meal causes health issues in piglets due to the presence of antigenic proteins. Although enzymatic treatment can break down antigenic proteins, subsequent high-temperature drying may impair amino acid (AA) digestibility via the Maillard reaction. This study evaluated whether the air-drying temperature affects the ileal AA digestibility of a novel reduced-sugar enzyme-treated soybean meal (ESM) in 25 kg pigs, using fishmeal as a high-digestibility reference. In two trials using pigs fitted with simple T-cannulas in the terminal ileum, ileal digestibility was assessed. In trial 1, a replicated 3 × 3 Latin square design with three diets (fishmeal, ESM, and a nitrogen-free diet; two pigs per diet) and three periods were used per square. Fishmeal showed greater apparent (82.50% vs. 45.01%) and standardized (86.60% vs. 48.86%) digestibility of crude protein and all amino acids than ESM dried at 130 °C. In trial 2, eight pigs were allocated to two diets in a two-period crossover design to compare the AA digestibility of ESM dried at high (130 °C; HtESM) and low (80 °C; LtESM) temperatures. LtESM exhibited greater apparent (82.24% vs. 52.40%) and standardized (86.37% vs. 56.47%) digestibility of crude protein and more amino acids than HtESM. Collectively, the drying temperature critically determined the AA digestibility of ESM, irrespective of its reducing sugar content.

## Simple Summary

Soybean meal is a major protein source for pigs, but it contains components that can harm young pigs’ digestion and cause diarrhea. While processing soybean meal with enzymes can improve its quality, a commonly used drying step using high heat might damage the nutrients by causing a chemical reaction (the Maillard reaction) between sugars and proteins. This study tested a new type of enzyme-treated soybean meal from which sugars were removed before processing. The ileal amino acid digestibility of enzyme-treated soybean meal (dried at high or low temperatures) was compared with that of high-quality fishmeal. It was observed that the low-temperature-dried enzyme-treated soybean meal allowed pigs to digest amino acids almost as well as fishmeal, and better than the high-temperature-dried soybean meal. These results demonstrate that drying temperature is a critical factor for enhancing the nutritional value of enzyme-treated soybean meal in pigs.

## 1. Introduction

Post-weaning diarrhea remains one of the most significant challenges in modern swine production. Its etiology is multifactorial, and a major contributing factor to this is the sudden dietary transition from the highly digestible milk consumed during lactation to a solid feed-based diet after weaning. Soybean meal, the primary plant-based protein source in swine diets, can cause hypersensitivity in weaning piglets, resulting in lower intestinal villus height and depressed growth performance when compared with dried skim milk [1]. Specifically, increasing the proportion of soybean meal in the diet, which raises dietary protein levels, has been linked to greater incidence of diarrhea and poorer growth performance [2,3,4].

The negative effects of soybean meal on weaned piglets are mainly caused by its high glycinin and β-conglycinin contents—two major antigenic proteins that, as shown by in vivo [5,6,7] and in vitro [8,9,10] studies, can damage the intestinal barrier (e.g., increased permeability), induce inflammation, and impair cellular function. To counteract the negative effects of soybean antigens on feed utilization and piglet growth performance, previous reviews have highlighted specific processing technologies, including extrusion, fermentation, and enzymatic treatment, that could reduce soybean protein immunoreactivity [11,12]. Notably, enzymatic treatment has drawn attention as an effective approach for simultaneously reducing anti-nutritional factors and improving gut health in piglets, possibly via enzyme-released small peptides [13,14,15].

Some studies have already analyzed the relationship between enzymatic hydrolysis parameters (e.g., enzyme type, reaction conditions) and the degradation efficiency of antigenic proteins [16,17]. However, in the enzymatic hydrolysis of soybean meal, the following challenges still occur: The soluble carbohydrates (e.g., raffinose and stachyose) in soybean meal may sterically hinder protease activity, potentially impairing the enzymatic hydrolysis of soybean proteins [18]. Water-soluble sugars such as raffinose and stachyose in soybean meal may participate in the Maillard reaction with amino acids during the drying process, reducing amino acid (AA) digestibility [19]. The crude protein (CP) content of enzymatically treated soybean meal is approximately 50% [20], while top-grade fishmeal typically contains more than 65% crude protein, creating a nutritional gap in fishmeal substitution. Further research is needed to develop deep-processing techniques for soybean meal to enhance its nutritional value.

To address these issues, a novel soybean meal processing method has been devised, which incorporates a “washing” step to remove water-soluble carbohydrates prior to enzymatic (protease) hydrolysis. It was hypothesized that this approach will result in a higher level of amino acids and lower content of reducing sugars. Moreover, when the enzymatically (protease) hydrolyzed soybean meal is subjected to air drying, this may have no negative effects on ileal AA digestibility.

## 2. Materials and Methods

### 2.1. Production of Enzyme-Treated Soybean Meal

**Figure 1:**
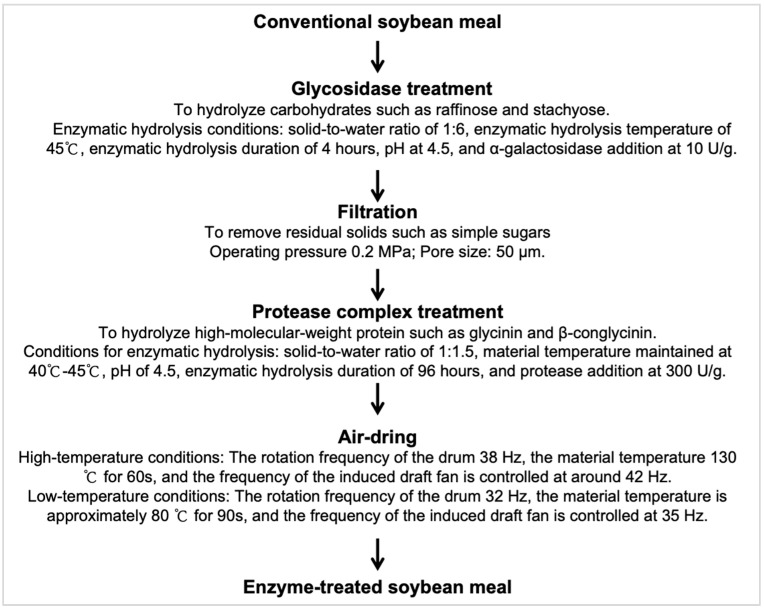
Production flow diagram of enzyme-treated soybean meal. α−Galactosidase activity unit is defined as the amount of enzyme required to release 1 μmol of p−nitrophenol per minute from a 5 mmol/L solution of p-nitrophenyl-α-D-galactopyranoside at 37 °C and pH 5.5. Protease activity unit is defined as the amount of protease that hydrolyzes casein to produce 1 μg of tyrosine per minute under specified temperature and pH conditions.

The enzyme-treated soybean meal (ESM) used in this study was processed and provided by Sichuan Runge Biotechnology Co., Ltd. (Deyang, China). In brief, soybean meal was subjected to α-glycosidase (10 U/g) treatment at pH 4.5, a solid-to-water ratio of 1:6, 45 °C, and 4 h to hydrolyze water-soluble carbohydrates, releasing simple sugars. The resulting mixture was filtered to remove residual solids, then treated with a protease complex (300 U/g) at a solid-to-water ratio of 1:1.5, pH 4.5, 40–45 °C, and 96 h to hydrolyze high-molecular-weight protein components, including glycinin and β-conglycinin. Subsequently, the enzymatically processed soybean meal was air-dried under controlled high-temperature (130 °C for 60 s) or low-temperature (80 °C for 90 s) conditions to obtain the final ESM products (Figure 1). A detailed compositional analysis of ESM is presented in Table 1.

### 2.2. Animals and Housing

Using pigs fitted with simple T-cannulas at the terminal ileum as the experimental model, two ileal amino acid digestibility trials were performed. The animal trials were carried out at the research center of the Animal Nutrition Institute, Sichuan Agricultural University. All experimental protocols were approved by the Animal Care and Use Committee of Sichuan Agricultural University (Approval No. SICAU–20230856). Crossbred barrows (Landrace × Large White × Duroc) with an average body weight of approximately 24.8 ± 1.85 kg were surgically implanted with simple T-cannulas at the terminal ileum. The surgical procedure was performed as previously described in [21]. In brief, the pigs were fasted for 48 h prior to surgery, during which they had ad libitum access to a 4.5% glucose solution administered orally after surgery to meet their basal energy requirements. Anesthesia was induced via an intramuscular injection of Zoletil^®^ (Virbac, Carros, France, 2 mL) and maintained through isoflurane–oxygen inhalation via an anesthesia ventilator. A 4–6 cm flank incision was made caudal to the last rib on the right abdominal wall. After the ileocecal junction was identified, an incision was made in the intestinal wall, and a T-cannula was surgically implanted. The cannula was exteriorized through a separate stab incision located 3 cm posterior to the rib cage. Prophylactic antibiotics (cephalosporins) were administered systemically for 72 h, and the surgical site was treated topically with erythromycin ointment to promote wound healing. Following a 14-day recovery period, during which the animals’ normal feeding behavior and clinical health were closely monitored, the experimental trials commenced. Throughout this study, the environmental conditions in the facility were maintained at 20 ± 2 °C with a relative humidity of 60 ± 10%. The animals were individually housed in stainless-steel metabolism cages (0.9 × 1.8 × 0.9 m) equipped with nipple drinkers to ensure ad libitum access to water.

### 2.3. Study Design and Diets

In trial 1, the objective was to investigate the ileal AA digestibility of fishmeal and ESM. Six crossbred barrows (Landrace × Large White × Duroc) fitted with T-cannulas and with body weights of 25.0 ± 2.0 kg were randomly allocated to three dietary treatments in a replicated 3 × 3 Latin square design, including a fishmeal diet, an ESM diet, and a nitrogen-free diet. Each diet was fed to two pigs, and the experiment consisted of three periods.

The diets were formulated with either fishmeal or ESM as the sole protein source, and corn starch was used as the primary energy source (Table 2 and Table 3). The ESM was air-dried for 60 s at 130 °C, a commonly used temperature for drying. All protein-containing diets were formulated to contain approximately 14–15% CP (Table 4), which is in line with the protein requirements of growing pigs under practical production conditions. To determine the endogenous protein and AA losses, a nitrogen-free diet was specifically designed. All diets were supplemented with adequate vitamins and minerals to meet the nutritional requirements of growing pigs according to NRC (2012) [22]. Moreover, 0.4% chromic oxide was added to each experimental diet as an exogenous marker for determining ileal AA digestibility. The experiment featured a three-period design, resulting in six replicates. Each period lasted for 7 days, consisting of a 5–day dietary adaptation phase and a 48 h ileal digesta collection phase (days 6–7). At the start of each experimental period, the pigs were weighed, and feed was provided at 4% of their body weight. The daily feed was divided into two equal meals, which were offered at 08.00 and 16.00 h.

Vitamin–mineral premix provided per kilogram of complete diet: 3500 IU Vitamin A; 400 IU Vitamin D_3_; 25.0 IU Vitamin E; 1.0 mg Vitamin K_3_; 5.7 mg Vitamin B_1_; 11.25 mg Vitamin B_2_; 6.0 mg Vitamin B_6_; 0.05 mg Vitamin B_12_; 60 mg niacin; 20.5 mg pantothenic acid; 0.6 mg folic acid; 0.2 mg biotin; 100 mg iron; 5 mg copper; 80 mg zinc; 3 mg manganese; 0.14 mg iodine; 0.25 mg selenium.

Trial 2 aimed to compare the ileal AA digestibility of high-temperature-dried (130 °C, 60 s) enzyme-treated soybean meal (HtESM) and low-temperature-dried (80 °C, 90 s) enzyme-treated soybean meal (LtESM). Eight crossbred barrows (Landrace × Large White × Duroc), with an average body weight of 24.5 ± 2.0 kg, were randomly assigned to two dietary treatments, including diets containing the HtESM or LtESM as the sole protein source. Corn starch was the primary energy source. The AA compositions of dietary ingredients and experimental diets are detailed in Table 5, Table 6 and Table 7. Endogenous protein and AA losses were corrected using data derived from trial 1. To facilitate ileal digestibility determination, 0.4% chromic oxide was incorporated into each experimental diet as an exogenous digestibility marker. The experiment employed a two-period crossover design, yielding eight replicates per treatment group.

Vitamin–mineral premix provided per kilogram of complete diet: 3500 IU Vitamin A; 400 IU Vitamin D_3_; 25.0 IU Vitamin E; 1.0 mg Vitamin K_3_; 5.7 mg Vitamin B_1_; 11.25 mg Vitamin B_2_; 6.0 mg Vitamin B_6_; 0.05 mg Vitamin B_12_; 60 mg niacin; 20.5 mg pantothenic acid; 0.6 mg folic acid; 0.2 mg biotin; 100 mg iron; 5 mg copper; 80 mg zinc; 3 mg manganese; 0.14 mg iodine; 0.25 mg selenium.

### 2.4. Sample Collection and Processing in the Amino Acid Digestibility Trial

Ileal digesta samples were collected on days 6 and 7 of each experimental period using a standardized protocol. Immediately following the morning meal (08.00 h), sample collection bags were securely attached to the T-cannulas. Digesta were collected continuously from 08.00 to 20.00 h, with bags replaced every 30 min or upon reaching capacity to ensure sample integrity. Collected digesta were immediately frozen at −20 °C to inhibit microbial degradation and preserve sample quality. Within each experimental period, individual pig samples were pooled, homogenized, and subsampled for lyophilization (freeze drying). The resulting lyophilized powder was ground through a 1 mm sieve and stored in airtight containers at temperatures below −20 °C until subsequent chemical analysis.

### 2.5. Chemical Analysis for Diet and Ileal Digesta Samples

The chemical compositions of experimental diets and ileal digesta samples were analyzed using standardized Association of Official Analytical Collaboration (AOAC) methods. Dry matter was determined gravimetrically following AOAC Official Method 930.15 (2007) [23]. Chromium concentrations in both diets and ileal digesta were quantified using AOAC Method 990.08 (2007) [23], while the CP content was measured via the Kjeldahl method (Method 984.13; 2007) [23] using an automated Kjeltec 8400 system (Foss Analytical, Hillerød, Denmark). Total nitrogen content was determined, and CP was calculated using the standard conversion factor N × 6.25. The ether extract (crude fat) content in the ingredients was determined using the Soxhlet extraction method (Method 922.06; AOAC 2007) [23].

Neutral detergent fiber (NDF), acid detergent fiber (ADF), and crude fiber (CF) were analyzed using Ankom Technology Methods, using an automatic fiber analyzer (Ankom Technology, Macedon, NY, USA). The ash content was measured via combustion in a muffle furnace (Method 961.14; AOAC 2007) [23], and starch content was quantified using the glucoamylase procedure (Method 979.10) [23]. Dietary fiber, defined as edible carbohydrate polymers with three or more monomeric units that are resistant to digestion and absorption in the small intestine due to the lack of endogenous enzymes, comprises two main fractions: soluble dietary fiber (SDF) and insoluble dietary fiber (IDF). In this study, both SDF and IDF were quantified using AOAC Official Method 991.43 [23], and the total dietary fiber (TDF) was calculated as the sum of SDF and IDF. The AA composition in feed ingredients, experimental diets, and ileal digesta was analyzed using AOAC Method 994.12 (2007) [23] using a Hitachi L-8900 high-performance amino acid analyzer (Hitachi High-Tech, Tokyo, Japan). Prior to analysis, samples underwent acid hydrolysis in 6 mol/L HCl containing phenol at 110 ± 2 °C for 24 h, followed by post-column ninhydrin derivatization. For sulfur-containing amino acids (methionine and cysteine), an additional oxidation step was performed using performic acid–phenol (0 °C, 16 h) prior to hydrolysis to ensure complete derivatization. Tryptophan was quantified separately via HPLC (Waters e2695, Waters Corporation, Santa Clara, CA, USA) following alkaline hydrolysis with lithium hydroxide (110 °C, 22 h; Method 982.30 E(c), 2007) [23] to prevent thermal degradation. The concentrations of glycinin (Catalog: 11S-EA) and β-conglycinin (Catalog: 7S-EA) in soybean meal and enzyme-treated soybean meal were quantified using commercial ELISA kits (Tianjin Longke Xinyu Biotechnology Co., Ltd., Tianjin, China) according to the manufacturer’s protocols.

### 2.6. Calculations

The standardized ileal digestibility (SID) and apparent ileal digestibility (AID) of CP and amino acids were assessed through the indicator method. According to the method previously described [24], the AID of dietary CP or AA was derived using a specific mathematical formula:

$$\mathrm{AID}\%=\left(1-\frac{\mathrm{Cr}\;\mathrm{in}\;\mathrm{diet}(\%)}{\mathrm{CP}\;\mathrm{or}\;\mathrm{AA}\;\mathrm{in}\mathrm{diet}(\%)}\times \frac{\mathrm{CP}\;\mathrm{or}\;\mathrm{AA}\;\mathrm{in}\;\mathrm{digesta}(\%)}{\mathrm{Cr}\;\mathrm{in}\;\mathrm{digesta}(\%)}\right)\times 100$$

Additionally, basal endogenous losses were estimated based on dry matter intake (DMI) using a dedicated calculation approach:

$$\mathrm{Basal}\;\mathrm{endogenous}\;\mathrm{losse}=\mathrm{CP}\;\mathrm{or}\;\mathrm{AA}\;\mathrm{in}\;\mathrm{digesta}\times \frac{\mathrm{Cr}\;\mathrm{in}\;\mathrm{diet}}{\mathrm{Cr}\;\mathrm{in}\;\mathrm{digesta}}$$

Finally, the SID values for CP and AA in the diets were determined through the following equation:

SID=AID+(Basal endogenous losse÷CP or AA in diet)

### 2.7. Statistical Analyses

The experimental data were analyzed using the MIXED procedure in SAS 9.4 (SAS Inst. Inc., Cary, NC, USA). The statistical model included the experimental diets as a fixed effect and the experimental period as a random effect. Since the experimental period had no significant impact on nutrient digestibility, the final model retained only the dietary treatment as the main effect. Multiple comparisons among groups were conducted using Duncan’s method for variance analysis. Statistical significance was declared at *p* < 0.01.

## 3. Results

### 3.1. Chemical Compositions of Ingredients and Diets

Enzymatic treatment of soybean meal induced changes in its chemical composition (Table 1). Compared to untreated soybean meal, the enzyme-treated soybean meal exhibited lower levels of fiber fractions, including CF, NDF, IDF, SDF, and TDF, as well as an increased CP content. Notably, concentrations of the primary soybean allergenic proteins, glycinin and β-conglycinin, were markedly reduced from 69.70 mg/g and 128.00 mg/g in soybean meal to 14.20 mg/g and 4.81 mg/g in ESM, respectively.

### 3.2. Apparent Ileal Digestibility of Crude Protein and Amino Acids for Fishmeal and Enzyme-Treated Soybean Meal in Pigs in Trial 1

As shown in Table 8, the apparent ileal digestibility of CP was greater in fishmeal (82.50%) than in enzyme-treated soybean meal (45.01%, *p* < 0.01). Furthermore, all measured amino acids, including arginine, histidine, isoleucine, leucine, lysine, methionine, phenylalanine, threonine, tryptophan, valine, alanine, aspartic acid, cystine, glutamic acid, glycine, serine, and tyrosine, exhibited greater apparent ileal digestibility in fishmeal compared to enzyme-treated soybean meal (*p* < 0.01 for all comparisons).

### 3.3. Standardized Ileal Digestibility of Crude Protein and Amino Acids for Fishmeal and Enzyme-Treated Soybean Meal in Pigs in Trial 1

After adjusting for endogenous losses using a nitrogen-free diet, the standardized ileal digestibility of amino acids was calculated (Table 9). The standardized ileal digestibility of CP was greater in fishmeal (86.60%) than in enzyme-treated soybean meal (48.86%, *p* < 0.01). Similarly, both essential and non-essential amino acids demonstrated greater standardized ileal digestibility in fishmeal than in enzyme-treated soybean meal (*p* < 0.01 for all comparisons).

### 3.4. Apparent Digestibility of Crude Protein and Amino Acids Between HtESM and LtESM in Trial 2

As shown in Table 10, the apparent digestibility of CP was lower in HtESM (52.40%) compared to LtESM (82.24%, *p* < 0.01). Furthermore, all measured amino acids (except for cystine), including arginine, histidine, isoleucine, leucine, lysine, methionine, phenylalanine, threonine, tryptophan, valine, alanine, aspartic acid, glutamic acid, glycine, proline, serine, and tyrosine, demonstrated greater apparent ileal digestibility in LtESM than in HtESM (*p* < 0.01 for all comparisons).

### 3.5. Standardized Ileal Digestibility of Crude Protein and Amino Acids Between HtESM and LtESM in Trial 2

The standardized ileal digestibility of CP and amino acids in HtESM and LtESM is presented in Table 11. The standardized ileal digestibility of CP was greater in LtESM (86.37%) than in HtESM (56.47%, *p* < 0.01). Additionally, the standardized ileal digestibility of arginine, histidine, isoleucine, leucine, lysine, methionine, phenylalanine, threonine, tryptophan, valine, alanine, aspartic acid, glutamic acid, glycine, proline, serine, and tyrosine, were greater in LtESM than in HtESM (*p* < 0.01). The standardized ileal digestibility of cystine in HtESM was similar to that in LtESM (*p* = 0.707).

The endogenous losses of amino acids (%) were as follows: arginine 0.02, histidine 0.02, isoleucine 0.02, leucine 0.03, lysine 0.02, methionine 0.01, phenylalanine 0.02, threonine 0.03, valine 0.02, alanine 0.03, aspartic acid 0.04, cysteine 0.01, glutamic acid 0.04, glycine 0.06, proline 0.07, serine 0.03, and tyrosine 0.01.

## 4. Discussion

In practical swine production, advanced processing of soybean meal primarily serves two critical objectives: to mitigate antinutritional factors, particularly glycinin and β-conglycinin, to minimize their detrimental effects on piglet intestinal health and prevent post-weaning stress syndrome [16,25]; and to increase nutritional value through targeted enzymatic hydrolysis, thereby enabling the partial substitution of costly protein sources such as fishmeal [26]. This approach can reduce feed production costs and contribute to sustainable swine production.

The efficacy of enzymatic hydrolysis is conventionally assessed through two primary indicators: (1) the reduction in soybean antigen content and (2) the improvement of AA digestibility. While extensive research has examined enzymatically hydrolyzed soybean meal [27,28,29,30,31], this study introduces a novel pre-hydrolysis carbohydrate removal step designed to specifically reduce soluble reducing sugars (e.g., raffinose and stachyose). This approach may minimize Maillard reaction products during the drying process by decreasing reducing sugar content, thereby preserving AA quality, and increase the AA concentration in enzyme-treated soybean meal, thereby enhancing its potential to replace fishmeal in swine diets. Chemical composition analyses confirmed the effectiveness of this processing method, which reduced soybean antigen content and increased CP content (approaching 60%). These findings confirm that the processing method successfully achieved its dual objectives of increasing CP content and minimizing undesirable soybean antigen levels. To determine nutritional value and ingredient substitution potential, the standardized ileal AA digestibility of enzyme-treated soybean meal and fishmeal was analyzed. Given that a lower level of dietary sugars may decrease Maillard reactions and enhance AA digestibility [19], the impact of this processing procedure on post-enzymatic hydrolysis AA digestibility was evaluated.

Since ESM has the potential to replace fishmeal in swine diets [15], the ileal amino acid digestibility between fishmeal and ESM was initially investigated. In Trial 1, the apparent ileal digestibility of CP and AA in ESM was lower than that in fishmeal. Regarding fishmeal, it was determined to be of a top grade based on the Chinese national standard for feed material and fishmeal (GB/T 19164-2021) [32]. Digestibility assays revealed that fishmeal exhibited exceptionally high CP standardized ileal digestibility (86.6%), and the measured standardized ileal digestibility of essential amino acids in this study was an average 91.28%. This value is in close agreement with the results obtained in growing pigs (87.78%) [33] and weaning pigs (90.08%) [28]. Notably, the SID of lysine is 93.7% for fishmeal in this study, which is consistent with the results of a more recent study [34]. This study examined 10 different sources of fishmeal and found an overall SID for lysine of 91.2%. Among them, four top-grade fishmeals (with a crude protein > 66%) exhibited an SID for lysine ranging from 90.2% to 95.9% [34]. Such consistency indicates that the analytical and calculation methods are reliable and that the current results are comparable to those from well-established international studies.

In contrast, the enzyme-treated soybean meal showed substantially lower standardized ileal digestibility values for essential amino acids, with the following coefficients: arginine (56.89%), histidine (56.26%), isoleucine (71.94%), leucine (73.97%), lysine (69.66%), methionine (77.42%), phenylalanine (76.43%), threonine (58.29%), and valine (66.74%). The standardized ileal digestibility observed in this enzyme-treated soybean meal was lower than that reported in other laboratory studies [20,22,28,35], as well as for conventional soybean meal evaluated in growing pigs [21,22,36,37]. These results clearly demonstrated the inferior nutritional quality of this enzyme-treated soybean meal compared to fishmeal, particularly in terms of essential AA bioavailability.

It should be noted that the CP content and AA composition of the diets used for amino acid digestibility evaluation in Trial 1 varied. The fishmeal diet (13.2% CP, 0.97% lysine) and the enzyme-treated soybean meal diet (14.03% CP, 0.76% lysine) differed in CP (by 0.83 percentage points) and lysine (by 0.21 percentage points) contents in the present study. It was speculated that the differences in dietary CP content and AA composition observed in Trial 1 are unlikely to affect the ileal amino acid digestibility of the test ingredients. This can be supported by a recent study which showed that increasing dietary CP from 7.06% to 11.01% and varying amino acid levels (e.g., lysine from 0.23% to 0.77%) did not affect the standardized ileal digestibility (SID) of amino acids in corn [38]. Similarly, in studies with soybean meal, differences in dietary CP (19.68% vs. 22.47%) and lysine (1.13% vs. 1.57%) did not influence the SID of amino acids in soybean meal when evaluated in growing pigs [38]. On the other hand, it was demonstrated that when cornstarch-based diets were formulated using soybean meal as the sole amino acid source and tested across six graded dietary CP levels, the apparent ileal digestibility of amino acids increased sharply from 4% to 16% CP and then plateaued between 16% and 24% CP [39]. Thus, this study cannot entirely rule out the potential impact of dietary CP level variations on amino acid digestibility.

The entire process in Trial 1 was carefully examined, and it was hypothesized that the drying temperature (130 °C) might be the underlying cause, as supported by the existing literature. Specifically, prolonged heating (e.g., 125 °C autoclaving for 30 min) linearly reduces AA digestibility, likely due to Maillard reaction products [40]. Notably, limited research has systematically examined the impact of drying temperature on the standardized ileal digestibility of amino acids in ESM. To address this knowledge gap, trial 2 was specifically designed to compare the ileal AA digestibility of enzyme-treated soybean meal processed at two distinct drying temperatures: 80 °C (LtESM) and 130 °C (HtESM). Analyses revealed that LtESM exhibited a higher lysine-to-CP ratio than HtESM, indicating a possible reduction in Maillard reaction products compared to HtESM [40]. The glycinin content in HtESM and LtESM was 17.4 mg/g and 16.5 mg/g, respectively, while the β-conglycinin content was 7.79 mg/g and 14.3 mg/g, respectively. In contrast, conventional soybean meal contained greater levels of glycinin (69.7 mg/g) and β-conglycinin (128 mg/g), indicating that the enzymatic treatment process effectively reduced the allergenic protein content in soybean meal. Animal trials confirmed that LtESM had greater standardized ileal digestibility values for both CP and amino acids than HtESM. However, trial 2 had a two-period crossover design, which has some limitations when applied in animal nutrition or physiology studies. In particular, when the number of experimental units (i.e., animals) is limited, such designs may exhibit lower statistical robustness compared to fully balanced designs (e.g., randomized complete block design or Latin Square design). This is especially relevant in cases where there is considerable individual variability among animals, which is common among pigs fitted with T-cannulas and can increase the risk of reaching incorrect conclusions or failing to properly test the study hypotheses.

The average standardized ileal digestibility of essential amino acids in LtESM was 89.56%, and the SID of crude protein was 86.37%. These values are comparable to or exceed those reported in previous studies, including the value presented by NRC (2012) (average SID of essential AA = 88.3%) [22] and other reports (average SID of essential AA = 90.08%) [28], as well as findings on fermented soybean meal, where the SID of essential AAs ranged between 84.3% and 88.8% [28,41,42].

Notably, when HtESM from trial 2 was compared with that from trial 1, the standardized ileal digestibility of CP was 56.82%, which was higher than the data of 48.86% recorded in trial 1. For essential amino acids, except for histidine (with SID values of 56.26% in trial 1 and 71.19% in trial 2), the standardized ileal digestibility values were highly consistent between the two trials. Of particular significance, the LtESM exhibited a high CP standardized ileal digestibility of 86.37%, which was close to the value of 86.60% recorded for fishmeal in trial 1. These results indicated that the LtESM had high ileal AA digestibility.

Although it was hypothesized that differences in Maillard reaction intensity contributed to the observed variations in ileal amino acid digestibility between drying temperatures in this study, the Maillard reaction severity (e.g., via reactive lysine content) could not be directly assessed due to analytical limitations. Notwithstanding this constraint, this study’s findings unequivocally reaffirm that drying temperature plays a pivotal role in determining the amino acid digestibility of enzyme-treated soybean meal. These research results offer valuable guidance for the advanced processing technologies for feed ingredients rich in amino acids.

The enzyme-treated soybean meal used in this study was priced at RMB 3000/ton more than conventional soybean meal. Nevertheless, even with this difference, enzyme-treated soybean meal remains more economical than fishmeal within the Chinese market. The low antigenic protein content and increased levels of CP and AA in enzyme-treated soybean meal highlight its suitability as a partial substitute for fishmeal, while its cost-effectiveness further enhances its practical applicability. Further research is needed to evaluate the effects of replacing fishmeal with low-temperature-dried, enzyme-treated soybean meal on the growth performance of piglets.

## 5. Conclusions

This study conclusively demonstrates that performing pre-treatment to reduce the water-soluble reducing sugar content prior to the enzymatic (protease) treatment of soybean meal effectively decreases the levels of antigenic proteins (glycinin and β-conglycinin). However, this approach does not alleviate the adverse impact of high-temperature drying on the ileal amino acid digestibility of enzyme-treated soybean meal. The findings highlight a critical processing parameter: the drying temperature following enzymatic (protease) treatment. This parameter should be prioritized as a key factor in enhancing the nutritional value of enzyme-treated soybean meal.

## Figures and Tables

**Table 1:** Compositions of soybean meal and enzyme-treated soybean meal (as-fed basis).

	Soybean Meal	Enzyme-Treated Soybean Meal
Dry matter, %	85.70	90.63
Crude protein, %	42.83	56.80
Neutral detergent fiber, %	12.98	7.02
Acid detergent fiber, %	11.14	6.64
Crude fiber, %	6.41	2.80
Crude fat, %	2.21	0.37
Crude ash, %	5.96	9.10
Starch, g/kg	44.80	26.20
Soluble dietary fiber, %	2.64	1.64
Insoluble dietary fiber, %	22.56	14.88
Total dietary fiber, %	25.20	16.52
Glycinin, mg/g	69.70	14.20
β-Conglycinin, mg/g	128.00	4.81

**Table 2:** Composition of diets for pigs in trial 1 (%, as-fed basis).

	Fishmeal	Enzyme-Treated Soybean Meal
Corn starch	66.92	58.17
Fishmeal	20.00	-
ESM	-	27.00
Soybean oil	2.00	2.00
Sucrose	10.00	10.00
Choline chloride	0.10	0.10
Chromic oxide	0.40	0.40
Limestone	-	0.80
Dicalcium phosphate	-	0.95
Sodium chloride	0.40	0.40
Vitamin–mineral premix	0.18	0.18
Total	100.00	100.00

ESM, enzyme-treated soybean meal.

**Table 3:** Compositions of amino acids in fishmeal and enzyme-treated soybean meal in trial 1 (%, as-fed basis).

	Fishmeal	Enzyme-Treated Soybean Meal
Dry matter	91.87	92.48
Indispensable amino acid		
Arginine	3.81	3.65
Histidine	2.02	1.34
Isoleucine	2.61	2.00
Leucine	4.71	3.46
Lysine	4.91	3.15
Methionine	1.96	0.64
Phenylalanine	2.70	2.31
Threonine	2.74	1.78
Tryptophan	0.67	0.76
Valine	2.97	2.15
Dispensable amino acid		
Alanine	4.08	3.33
Aspartic acid	5.91	5.38
Cystine	0.67	0.64
Glutamic acid	8.51	8.96
Glycine	3.90	5.77
Proline	2.60	4.47
Serine	2.56	2.18
Tyrosine	2.25	1.39
Total amino acids	59.58	53.36

**Table 4:** Chemical analyses of experimental diets in trial 1 (%, as-fed basis).

	Fishmeal	Enzyme-Treated Soybean Meal
Dry matter	91.41	90.97
Crude protein	13.20	14.03
Indispensable amino acid		
Arginine	0.79	0.84
Histidine	0.34	0.21
Isoleucine	0.54	0.48
Leucine	0.98	0.87
Lysine	0.97	0.76
Methionine	0.28	0.15
Phenylalanine	0.55	0.59
Threonine	0.57	0.41
Tryptophan	0.15	0.21
Valine	0.59	0.55
Dispensable amino acid		
Alanine	0.84	0.95
Aspartic acid	1.16	1.24
Cystine	0.11	0.23
Glutamic acid	1.75	2.22
Glycine	0.79	1.51
Proline	0.65	1.30
Serine	0.50	0.46
Tyrosine	0.31	0.29

**Table 5:** Analyses of the compositions of amino acid contents of two enzyme-treated soybean meal samples in trial 2 (%, as-fed basis).

	HtESM	LtESM
Dry matter	93.50	93.40
Crude protein	59.89	60.40
Indispensable amino acid		
Arginine	3.73	3.86
Histidine	1.31	1.33
Isoleucine	2.34	2.36
Leucine	4.05	4.05
Lysine	3.97	4.16
Methionine	1.14	1.24
Phenylalanine	2.65	2.64
Threonine	2.07	2.07
Tryptophan	0.74	0.76
Valine	2.44	2.49
Dispensable amino acid		
Alanine	3.16	3.21
Aspartic acid	6.21	6.20
Cystine	0.65	0.64
Glutamic acid	10.43	10.47
Glycine	4.45	4.57
Proline	4.46	4.63
Serine	2.56	2.61
Tyrosine	1.72	1.72
Total amino acids	57.34	58.25
glycinin, mg/g	17.40	16.50
β-conglycinin, mg/g	7.79	14.30

HtESM, high-temperature-dried enzyme-treated soybean meal; LtESM, low-temperature-dried enzyme-treated soybean meal.

**Table 6:** Composition of experimental diets for pigs in trial 2 (%, as-fed basis).

	HtESM	LtESM
Corn starch	63.67	63.67
HtESM	21.50	-
LtESM	-	21.50
Soybean oil	2.00	2.00
Sucrose	10.00	10.00
Choline chloride	0.10	0.10
Chromic oxide	0.40	0.40
Limestone	0.80	0.80
Dicalcium phosphate	0.95	0.95
Sodium chloride	0.40	0.40
Vitamin–mineral premix	0.18	0.18
Total	100.00	100.00

HtESM, high-temperature-dried enzyme-treated soybean meal; LtESM, low-temperature-dried enzyme-treated soybean meal.

**Table 7:** Analyses of the compositions of amino acids of the experimental diets in trial 2 (%, as-fed basis).

	HtESM	LtESM
Dry matter	90.10	90.00
Crude protein	13.13	12.92
Indispensable amino acid		
Arginine	0.74	0.76
Histidine	0.23	0.24
Isoleucine	0.48	0.46
Leucine	0.85	0.83
Lysine	0.75	0.75
Methionine	0.24	0.25
Phenylalanine	0.51	0.55
Threonine	0.42	0.41
Tryptophan	0.17	0.19
Valine	0.53	0.58
Dispensable amino acid		
Alanine	0.69	0.70
Aspartic acid	1.27	1.27
Cystine	0.16	0.18
Glutamic acid	2.15	2.18
Glycine	0.93	0.96
Proline	0.83	0.83
Serine	0.51	0.53
Tyrosine	0.29	0.30

HtESM, high-temperature-dried enzyme-treated soybean meal; LtESM, low-temperature-dried enzyme-treated soybean meal.

**Table 8:** Apparent digestibility of crude protein and amino acids in fishmeal and enzyme-treated soybean meal in trial 1 (%).

	Fishmeal	Enzyme-Treated Soybean Meal	*p* Value
Crude protein	82.50 ± 1.17 ^A^	45.01 ± 3.90 ^B^	<0.01
Indispensable amino acid			
Arginine	93.08 ± 0.52 ^A^	55.03 ± 4.71 ^B^	<0.01
Histidine	86.31 ± 1.46 ^A^	49.17 ± 2.68 ^B^	<0.01
Isoleucine	88.26 ± 0.96 ^A^	68.83 ± 1.40 ^B^	<0.01
Leucine	89.61 ± 0.76 ^A^	71.32 ± 1.13 ^B^	<0.01
Lysine	91.88 ± 0.53 ^A^	67.31 ± 1.34 ^B^	<0.01
Methionine	90.50 ± 0.85 ^A^	73.12 ± 1.94 ^B^	<0.01
Phenylalanine	87.22 ± 0.91 ^A^	73.91 ± 1.10 ^B^	<0.01
Threonine	86.37 ± 1.06 ^A^	52.19 ± 1.84 ^B^	<0.01
Tryptophan	76.16 ± 3.53 ^A^	34.21 ± 5.46 ^B^	<0.01
Valine	86.79 ± 0.98 ^A^	63.26 ± 1.50 ^B^	<0.01
Dispensable amino acid			
Alanine	86.58 ± 1.06 ^A^	41.78 ± 3.65 ^B^	<0.01
Aspartic acid	83.07 ± 1.38 ^A^	54.02 ± 1.61 ^B^	<0.01
Cystine	78.91 ± 1.62 ^A^	50.22 ± 4.24 ^B^	<0.01
Glutamic acid	89.57 ± 0.86 ^A^	63.96 ± 1.44 ^B^	<0.01
Glycine	81.97 ± 1.42 ^A^	23.11 ± 3.79 ^B^	<0.01
Serine	86.20 ± 0.90 ^A^	57.67 ± 1.45 ^B^	<0.01
Tyrosine	83.35 ± 1.43 ^A^	68.51 ± 1.81 ^B^	<0.01

Values are means ± S.E. ^A,B^ denotes *p* < 0.01 using Duncan’s test method. *n* = 6.

**Table 9:** Standardized digestibility of crude protein and amino acids in fishmeal and enzyme-treated soybean meal in trial 1 (%).

	Fishmeal	Enzyme-Treated Soybean Meal	*p* Value
Crude protein	86.60 ± 1.17 ^A^	48.86 ± 3.90 ^B^	<0.01
Indispensable amino acid			
Arginine	95.07 ± 0.52 ^A^	56.89 ± 4.71 ^B^	<0.01
Histidine	90.70 ± 1.46 ^A^	56.26 ± 2.68 ^B^	<0.01
Isoleucine	91.03 ± 0.96 ^A^	71.94 ± 1.40 ^B^	<0.01
Leucine	91.98 ± 0.76 ^A^	73.97 ± 1.13 ^B^	<0.01
Lysine	93.74 ± 0.53 ^A^	69.66 ± 1.34 ^B^	<0.01
Methionine	92.82 ± 0.85 ^A^	77.42 ± 1.94 ^B^	<0.01
Phenylalanine	89.94 ± 0.91 ^A^	76.43 ± 1.10 ^B^	<0.01
Threonine	90.78 ± 1.06 ^A^	58.29 ± 1.84 ^B^	<0.01
Tryptophan	86.65 ± 3.53 ^A^	41.67 ± 5.46 ^B^	<0.01
Valine	90.04 ± 0.98 ^A^	66.74 ± 1.50 ^B^	<0.01
Dispensable amino acid			
Alanine	89.34 ± 1.06 ^A^	44.21 ± 3.65 ^B^	<0.01
Aspartic acid	86.06 ± 1.38 ^A^	56.80 ± 1.61 ^B^	<0.01
Cystine	86.20 ± 1.62 ^A^	53.69 ± 4.24 ^B^	<0.01
Glutamic acid	91.85 ± 0.86 ^A^	65.74 ± 1.44 ^B^	<0.01
Glycine	88.51 ± 1.42 ^A^	26.09 ± 3.79 ^B^	<0.01
Serine	90.82 ± 0.90 ^A^	62.67 ± 1.45 ^B^	<0.01
Tyrosine	86.16 ± 1.43 ^A^	71.51 ± 1.81 ^B^	<0.01

The endogenous losses of amino acids (%) were as follows: arginine 0.02, histidine 0.02, isoleucine 0.02, leucine 0.03, lysine 0.02, methionine 0.01, phenylalanine 0.02, threonine 0.03, valine 0.02, alanine 0.03, aspartic acid 0.04, cysteine 0.01, glutamic acid 0.04, glycine 0.06, proline 0.07, serine 0.03, and tyrosine 0.01. Values are means ± S.E. ^A,B^ denotes *p* < 0.01 using Duncan’s test method. *n* = 6.

**Table 10:** Apparent digestibility of crude protein and amino acids in HtESM and LtESM in trial 2 (%).

	HtESM	LtESM	*p* Value
Crude protein	52.40 ± 2.56 ^B^	82.24 ± 1.50 ^A^	<0.01
Indispensable amino acid			
Arginine	62.17 ± 2.69 ^B^	91.29 ± 0.79 ^A^	<0.01
Histidine	64.79 ± 1.15 ^B^	81.92 ± 1.79 ^A^	<0.01
Isoleucine	74.35 ± 1.09 ^B^	84.52 ± 1.27 ^A^	<0.01
Leucine	75.14 ± 0.95 ^B^	85.41 ± 1.15 ^A^	< 0.01
Lysine	72.35 ± 1.14 ^B^	87.79 ± 1.34 ^A^	<0.01
Methionine	88.69 ± 0.55 ^B^	93.97 ± 0.75 ^A^	<0.01
Phenylalanine	74.61 ± 1.20 ^B^	86.78 ± 0.92 ^A^	<0.01
Threonine	60.72 ± 1.44 ^B^	75.69 ± 2.13 ^A^	<0.01
Tryptophan	72.67 ± 2.46 ^B^	84.51 ± 1.63 ^A^	0.01
Valine	67.89 ± 1.40 ^B^	84.56 ± 1.27 ^A^	<0.01
Dispensable amino acid			
Alanine	42.48 ± 3.22 ^B^	82.52 ± 1.76 ^A^	<0.01
Aspartic acid	60.15 ± 1.93 ^B^	84.67 ± 1.13 ^A^	<0.01
Cystine	79.18 ± 1.73	80.94 ± 2.63	0.586
Glutamic acid	66.06 ± 1.92 ^B^	87.34 ± 2.39 ^A^	<0.01
Glycine	6.44 ± 6.86 ^B^	81.75 ± 1.51 ^A^	<0.01
Proline	12.22 ± 8.94 ^B^	73.39 ± 3.80 ^A^	<0.01
Serine	67.28 ± 1.45 ^B^	83.27 ± 1.36 ^A^	<0.01
Tyrosine	77.25 ± 0.78 ^B^	83.53 ± 1.23 ^A^	<0.01

HtESM, high-temperature-dried enzyme-treated soybean meal; LtESM, low-temperature-dried enzyme-treated soybean meal. Values are means ± S.E. ^A,B^ denotes *p* < 0.01 using Duncan’s test method. *n* = 8.

**Table 11:** Standardized digestibility of crude protein and amino acids in HtESM and LtESM in trial 2 (%).

	HtESM	LtESM	*p* Value
Crude protein	56.47 ± 2.56 ^B^	86.37 ± 1.50 ^A^	<0.01
Indispensable amino acid			
Arginine	64.26 ± 2.69 ^B^	93.32 ± 0.79 ^A^	<0.01
Histidine	71.19 ± 1.15 ^B^	88.05 ± 1.79 ^A^	<0.01
Isoleucine	77.43 ± 1.09 ^B^	87.73 ± 1.27 ^A^	<0.01
Leucine	77.83 ± 0.95 ^B^	88.16 ± 1.15 ^A^	<0.01
Lysine	74.72 ± 1.14 ^B^	90.16 ± 1.34 ^A^	<0.01
Methionine	91.35 ± 0.55 ^B^	96.52 ± 0.75 ^A^	<0.01
Phenylalanine	77.50 ± 1.20 ^B^	89.46 ± 0.92 ^A^	<0.01
Threonine	66.62 ± 1.44 ^B^	81.73 ± 2.13 ^A^	<0.01
Tryptophan	81.78 ± 2.46 ^B^	92.66 ± 1.63 ^A^	<0.01
Valine	71.46 ± 1.40 ^B^	87.81 ± 1.27 ^A^	<0.01
Dispensable amino acid			
Alanine	45.79 ± 3.22 ^B^	85.78 ± 1.76 ^A^	<0.01
Aspartic acid	62.84 ± 1.93 ^B^	87.36 ± 1.13 ^A^	<0.01
Cystine	84.11 ± 1.73	85.33 ± 2.63	0.707
Glutamic acid	67.89 ± 1.92 ^B^	89.14 ± 2.39 ^A^	<0.01
Glycine	11.92 ± 6.86 ^B^	87.04 ± 1.51 ^A^	<0.01
Proline	20.18 ± 8.94 ^B^	81.34 ± 3.80 ^A^	<0.01
Serine	71.75 ± 1.45 ^B^	87.57 ± 1.36 ^A^	<0.01
Tyrosine	80.22 ± 0.78 ^B^	86.40 ± 1.23 ^A^	<0.01

HtESM, high-temperature-dried enzyme-treated soybean meal; LtESM, low-temperature-dried enzyme-treated soybean meal. Values are means ± S.E. ^A,B^ denotes *p* < 0.01, using Duncan’s test method. *n* = 8.

## Data Availability

The data presented in this study are available from the corresponding author upon reasonable request.

## Abbreviations

The following abbreviations are used in this manuscript:

ESM	Enzyme-treated soybean meal
HtESM	High-temperature-dried enzyme-treated soybean meal
LtESM	Low-temperature-dried enzyme-treated soybean meal
AOAC	Association of Official Analytical Collaboration
SID	Standardized ileal digestibility
AID	Apparent ileal digestibility
CP	Crude protein
AAs	Amino acids
